# Optimizing Kappa-Deleting Recombination Excision Circles (KREC) Cut-Off Values in Russian Newborn Screening Program: Balancing Sensitivity and False-Positive Rates

**DOI:** 10.3390/ijns12030068

**Published:** 2026-08-19

**Authors:** Andrey Marakhonov, Anna Mukhina, Irina Efimova, Natalya Balinova, Yulia Rodina, Rena Zinchenko, Sergey Voronin, Anna Shcherbina, Sergey Kutsev

**Affiliations:** 1 Research Centre for Medical Genetics, Moscow 115522, Russia; ffmanya@yandex.ru (A.M.); efimova.geneticist@gmail.com (I.E.); balinovs@mail.ru (N.B.); renazinchenko@mail.ru (R.Z.); voroninsvvlad@mail.ru (S.V.); kutsev@mail.ru (S.K.); 2 Dmitry Rogachev National Medical Research Center of Pediatric Hematology, Oncology and Immunology, Moscow 117198, Russia; rodina.julija@rambler.ru (Y.R.); shcher26@hotmail.com (A.S.)

**Keywords:** newborn screening, kappa-deleting recombination excision circles (KREC), cut-off, X-linked agammaglobulinemia, transient B-cell lymphopenia, improved algorithm

## Abstract

Newborn screening (NBS) for primary immunodeficiencies (PIDs) increasingly incorporates kappa-deleting recombination excision circles (KREC) to enable early detection of B-cell lymphopenia, particularly agammaglobulinemia (AGG). However, optimal KREC cut-off values remain poorly defined, limiting sensitivity and contributing to high false-positive rates. We evaluated KREC-based NBS using data from a nationwide Russian program that screened more than 3.4 million newborns. KREC measurements from 66 patients with genetically confirmed AGG and 534 newborns with initially abnormal screening results but normal immunophenotyping were analyzed using statistical and ROC-based approaches. By using a KREC cut-off of 100 copies per 10^5^ cells in the first NBS sample and a KREC cut-off of 250 copies per 10^5^ cells after a polymerase chain reaction (PCR) test on a second dried blood spot (DBS) collected approximately 19 days later, the detection of classical and atypical X-linked AGG, other forms of AGG, and additional PIDs associated with B-cell lymphopenia was significantly improved. Retrospective analysis of a pilot cohort (n = 202,908) indicated that, if a KREC cut-off of 250 copies per 10^5^ cells was applied with the second PCR test, this adjustment would increase the proportion of screen-positive newborns to 2.75‰. To reduce the resulting referral burden, we assessed third KREC testing of a third DBS sample approximately 2 months after birth and showed that persistent KREC reduction distinguishes AGG from transient B-cell lymphopenia. We propose a revised three-step NBS algorithm incorporating a third PCR test with a cut-off of 850 copies per 10^5^ cells. This strategy reduces unnecessary referrals while maintaining diagnostic sensitivity and clinical safety, supporting implementation of adaptive, multistep NBS approaches.

## 1. Introduction

Early detection of inborn errors of immunity (IEI) has been revolutionized by population-wide newborn screening (NBS) utilizing the quantification of T-cell receptor excision circles (TRECs) in dried blood spots, enabling the presymptomatic identification of severe combined immunodeficiency (SCID) and other forms of severe T-cell lymphopenia [1]. SCID is included in the United States Recommended Uniform Screening Panel (RUSP) [2], reflecting a high level of evidence for clinical benefit, feasibility, and availability of effective early interventions [3]. However, TREC-based algorithms are intrinsically limited to T-lymphopoiesis and do not directly address profound B-cell defects that may be clinically silent at birth yet associated with substantial morbidity once maternally derived immunoglobulins wane [4].

Kappa-deleting recombination excision circles (KRECs) are episomal DNA by-products of immunoglobulin kappa light-chain rearrangement and serve as a surrogate marker of newly formed B cells. In multiplex quantitative PCR formats, KREC testing can be added to existing TREC workflows without major changes to specimen type or primary laboratory platform, thereby expanding NBS from severe T-cell lymphopenia to severe T- and B-cell lymphopenia in a single assay (TREC/KREC). Large pilot and implementation studies have demonstrated the operational feasibility of combined TREC/KREC screening and its ability to detect clinically relevant immune phenotypes beyond classical SCID, including syndromic T/B lymphopenias [5,6].

The primary rationale for KREC inclusion is the potential to identify congenital agammaglobulinemia (AGG)—most prominently X-linked agammaglobulinemia (XLA, BTK deficiency)—before severe bacterial infections occur. XLA is characterized by an early block in B-cell differentiation and very low or absent circulating B cells, a phenotype that is conceptually well-matched to KREC-based detection. Reviews of the IEI NBS experience have repeatedly highlighted XLA as the “best-known” B-cell disorder that can be detected at birth by KREC analysis, alongside rarer autosomal recessive forms of AGG [7]. At the same time, the policy landscape remains cautious: recent international summaries explicitly note that, in the United States, KREC has not been recommended by state programs or by RUSP, underscoring that the evidence base for routine population screening of B-cell disorders has not reached the maturity of SCID screening [8].

This caution reflects genuine controversy. First, the target condition debate is substantive: unlike SCID, many infants with XLA do not develop life-threatening infections in the neonatal period, raising questions about the incremental benefit of detecting XLA at birth versus early infancy [9]. Moreover, current treatment options for AGG, primarily immunoglobulin replacement therapy (IgRT), are not curative and do not achieve outcomes comparable to hematopoietic stem cell transplantation (HSCT) for SCID. Patients receiving IgRT remain susceptible to infections and may continue to experience substantial morbidity [10]. Nevertheless, additional therapeutic approaches, including gene therapy and HSCT, are currently being investigated for XLA [11,12,13,14]. The development of such treatment strategies may provide a stronger evidence-based rationale for including AGG in NBS programs. Second, the spectrum of disorders associated with low KRECs is broader than AGG; while this can be advantageous for case finding, it can also complicate follow-up pathways and family counseling when the screening phenotype is nonspecific or transient. These issues have driven heterogeneity across programs in testing algorithms (repeat testing, dual-threshold strategies, use of internal controls), recall criteria, and confirmatory immunophenotyping pathways [5,7].

Despite these concerns, several potential benefits motivate ongoing evaluation of KREC-based screening. Combined TREC/KREC testing can identify infants with severe B-cell lymphopenia who may benefit from anticipatory infection prevention, tailored vaccination decisions, early immunology referral, and timely initiation of immunoglobulin replacement when indicated. Moreover, real-world pilots have reported identification of congenital AGG cases through KREC-inclusive algorithms, supporting clinical plausibility for net benefit in at least a subset of screened populations [6,15]. In addition, multiplex approaches that include KRECs have been positioned as scalable platforms for expanding NBS targets where health systems are already investing in molecular first-tier assays [16].

A critical prerequisite for translating these theoretical and pilot-scale benefits into an effective public health program is rigorous local estimation of the KREC cut-off values. Cut-off selection is not a purely technical exercise; it operationalizes the trade-off between sensitivity for severe B-cell lymphopenia (including AGG) and the burden of false-positive referrals, repeat testing, and downstream diagnostics. Programs that have implemented TREC/KREC screening emphasize that standardization remains incomplete and that cut-offs frequently require empirical derivation from large newborn cohorts using specific assay chemistry, punch size, extraction method, calibration strategy, and population context of a given laboratory [17]. Accordingly, systematic cut-off estimation represents a necessary step toward improving screening effectiveness—maximizing true-case detection while maintaining acceptable specificity, referral rates, and follow-up capacity—and is foundational to any evidence-based discussion of whether and how KREC should be incorporated alongside TREC in routine NBS.

The effectiveness of NBS for AGG using KREC is highly dependent on the cut-off values applied within the screening program, as these thresholds determine the size and composition of the screening-positive group of newborns who subsequently undergo confirmatory diagnostic testing. However, there is no universally accepted KREC cut-off value for NBS. This issue is further compounded by the limited number of studies that have incorporated KREC into their screening design. In most remaining cases, KREC cut-off values were not established using rigorous statistical methodologies but were instead defined on the basis of general or pragmatic considerations. KREC values are also influenced by biological and pre-analytical factors (e.g., gestational age, leukocyte content, and blood spot quality), which can increase false-positive referrals if cut-offs are not appropriately calibrated to local populations and laboratory workflows [18].

In 2023, the Russian Federation initiated a nationwide newborn screening program that included TREC and KREC detection [19]. Since its implementation, more than 3 million newborns have been screened, leading to the identification of a substantial number of patients with B-cell lymphopenia. This study is devoted to a systematic large-scale evaluation of KREC performance and to the estimation of an optimal KREC cut-off value based on this dataset.

## 2. Materials and Methods

### 2.1. NBS Program and Diagnostic Testing

The nationwide NBS program was carried out as described elsewhere [19]. In summary, TREC/KREC-based screening was conducted starting 1 January 2023, and by December 2025, more than 3.5 million neonates had been screened. Written parental informed consent was obtained for all newborns enrolled in the NBS program.

Dried blood spot (DBS) samples were collected according to the national NBS protocol: on the 2nd day of life in term newborns and on the 7th day of life in preterm newborns. Preterm birth was defined according to the WHO criteria as delivery occurring before 37 completed weeks of gestation [20].

The established NBS algorithm comprises two PCR tests from different DBS samples, followed by immunophenotyping using flow cytometry in cases with decreased KREC levels and subsequent genetic analysis, including whole-exome sequencing (WES) [19] or whole-genome sequencing (WGS) as needed [21]. The median age at receipt of second PCR results for full-term newborns is 19 days (interquartile range: 15–25).

The first PCR was spread across 10 centers for NBS [22] using two commercial multiplex qPCR-based test systems: TK-SMA kit (ABV-Test LLC, Moscow, Russia) and NeoScreen 104 SMA/TREC/KREC REAL-TIME PCR Detection Kit (DNA-Technology TS, LLC, Moscow, Russia), and according to the manufacturers’ recommendations, a cut-off of 100 copies per 10^5^ cells was used. The second KREC tests were performed uniformly in duplicates at a single site, the Research Centre for Medical Genetics (Moscow, Russia), using newly collected DBS cards and the Eonis™ SCID-SMA kit (Wallac Oy, Turku, Finland) on the JANUS Extraction instrument (Perkin Elmer, Turku, Finland). Subsequently, real-time PCR analysis was performed using Applied Biosystems QuantStudio 5 Dx instruments (Thermo Fisher Scientific, Waltham, MA, USA), according to the manufacturer’s guidelines and recommendations. For the second PCR testing, an initial cut-off of 100 copies per 10^5^ cells was used; this threshold was subsequently revised during the study.

For samples which are screen-positive after the second PCR, subsequent immunological and genetic confirmation of the IEI diagnosis was performed as described earlier [6]. Briefly, immunophenotyping, which was repeated 1–2 months later, and genetic analysis were undertaken. Where available, a repeat KREC assessment was performed simultaneously with this follow-up flow cytometry. IEI diagnosis was established according to the European Society for Immunodeficiencies (ESID) diagnostic criteria [23] and the classification from the International Union of Immunological Societies Expert Committee [24]. SCID diagnosis was established according to the Primary Immune Deficiency Treatment Consortium (PIDTC) 2022 Definitions [25]. Clinical data for the current analysis were obtained from the Russian registry of IEIs [26]. These included clinical picture description and diagnosis.

### 2.2. Patient Groups for the Second KREC Test Cut-Off Determination and Validation

From the 3.5 million newborns who underwent NBS, KREC values obtained during the second PCR test from two groups (Case group and Control group) were analyzed to determine an optimal second PCR cut-off that balanced sensitivity and specificity.

Patients with genetically confirmed AGG were considered the true positives and formed the Case group. This group included 66 patients with different forms of AGG: XLA caused by *BTK* variants (n = 24), autosomal recessive *IGLL1*-related λ5 deficiency (n = 39), and rarer forms of AGG, including autosomal dominant *TCF3*-related AGG type 8A (n = 1), autosomal recessive *TCF3*-related AGG type 8B (n = 1), and autosomal recessive *IGHM*-related AGG type 1 (n = 1).

The Control group comprised newborns who were initially classified as screen-positive after the second KREC test but subsequently showed normal findings on flow cytometric immunophenotyping (n = 534). This group included newborns with secondary or transient B-cell lymphopenia that resolved within the first 1–2 months of life, as well as healthy newborns with normal B-cell counts.

Using the second KREC cut-off determined above, we subsequently estimated the expected increase in detection of other patient groups (Comparison group) who may present with low KREC values on NBS, typically but not invariably accompanied by reduced TREC values. The Comparison group included patients with T^−^B^−^ SCID (n = 22), Nĳmegen breakage syndrome (Nĳmegen syndrome) (n = 14), ataxia–telangiectasia syndrome (A–T) (n = 8), and trisomy 21 (n = 31), all identified through NBS of the above-mentioned 3.5 million newborns using the established protocol. This group was included in the analysis because these conditions may be associated with decreased KREC levels in NBS.

As the second PCR test was performed in duplicate according to the established protocol described above, most newborns had two KREC measurements. In total, we collected 130 KREC measurements from 66 patients with various forms of AGG, as well as 1042 measurements from 534 newborns in the Control group and 104 from the Comparison group.

The retrospective NBS cohort used for cut-off validation was derived from a previously published NBS pilot study in which newborns underwent TREC and KREC testing (n = 202,908). This cohort was considered a cross-sectional reference sample of the general newborn population, as it had been tested using the same qPCR kit as that employed for the second PCR test in the nationwide NBS program [6].

### 2.3. Statistical Analysis

Zero copies of TREC/KREC per 10^5^ cells were represented as 0.001 copies/10^5^ cells in figures as well as in statistical analysis.

The data collected during the study were analyzed using GraphPad Prism 10.4.0 (GraphPad Software, San Diego, CA, USA; https://www.graphpad.com). Throughout the analysis, data were presented as the median with interquartile range, unless specified otherwise. The maximum likelihood ratio in a receiver operating characteristic (ROC) analysis [27] was used to determine the optimal cut-off using GraphPad Prism 10.4.0 and the *pROC* package (version 1.18.5) [28] for R (version 4.3.2), run in RStudio 2025.05.0+496 (Posit Software, PBC, Boston, MA, USA; https://dailies.rstudio.com/version/2025.05.0+496.pro5/, assessed on 1 March 2026). To independently estimate the threshold, the thres2 function from the *ThresholdROC* package (version 2.9.4) [29,30,31] for R was also employed. Additionally, Fisher’s 95% confidence interval (95% CI) for proportional data was computed using the freeware statistical program WinPepi v. 11.65 [32] (http://www.brixtonhealth.com/pepi4windows.html, assessed on 1 March 2026).

## 3. Results

The main descriptive statistical characteristics of KREC values in the Case, Control, and Comparison groups are presented in Table 1. Across the AGG case group, KREC values were generally very low, with median values of 0 copies per 10^5^ cells in patients with XLA, λ5 deficiency, and rare forms of AGG. However, the upper range of KREC values showed considerable variability, including maximum values of 331.0 copies per 10^5^ cells in XLA, 333.4 copies per 10^5^ cells in λ5 deficiency, and 198.0 copies per 10^5^ cells in rare AGG. These findings indicate that, although most AGG patients had absent or markedly reduced KREC values, a first PCR cut-off of 100 copies per 10^5^ cells would not have suboptimal sensitivity when used as a cut-off for the second PCR test, since some true-positive AGG cases would have KREC values above this threshold.

Similar variability was also observed in the Comparison groups, including patients with T^−^B^−^ SCID, Nĳmegen syndrome, A–T, and trisomy 21. The median KREC value was 0 copies per 10^5^ cells in T^−^B^−^ SCID and Nijmegen syndrome but higher in A–T and trisomy 21, reaching 147.7 and 115.3 copies per 10^5^ cells, respectively. In the Control group, the median KREC value was 130.3 copies per 10^5^ cells, with a broad distribution extending to substantially higher values. Finally, Dunn’s multiple comparisons test demonstrated that only the Control group differed significantly from the XLA group, whereas the AGG subgroups and other groups did not show significant differences from XLA.

Of the 24 XLA patients, 6 (25.00%; 95% CI: 9.77–46.71%) had KREC values above the 100 copies per 10^5^ cells cut-off at the second PCR test stage (Table 2). Since XLA is one of the primary target disorders of KREC-based NBS, a threshold that misses a substantial proportion of affected patients would be insufficiently sensitive for screening purposes. This finding prompted further refinement of the optimal KREC cut-off value for the second PCR test stage.

**Figure 1:**
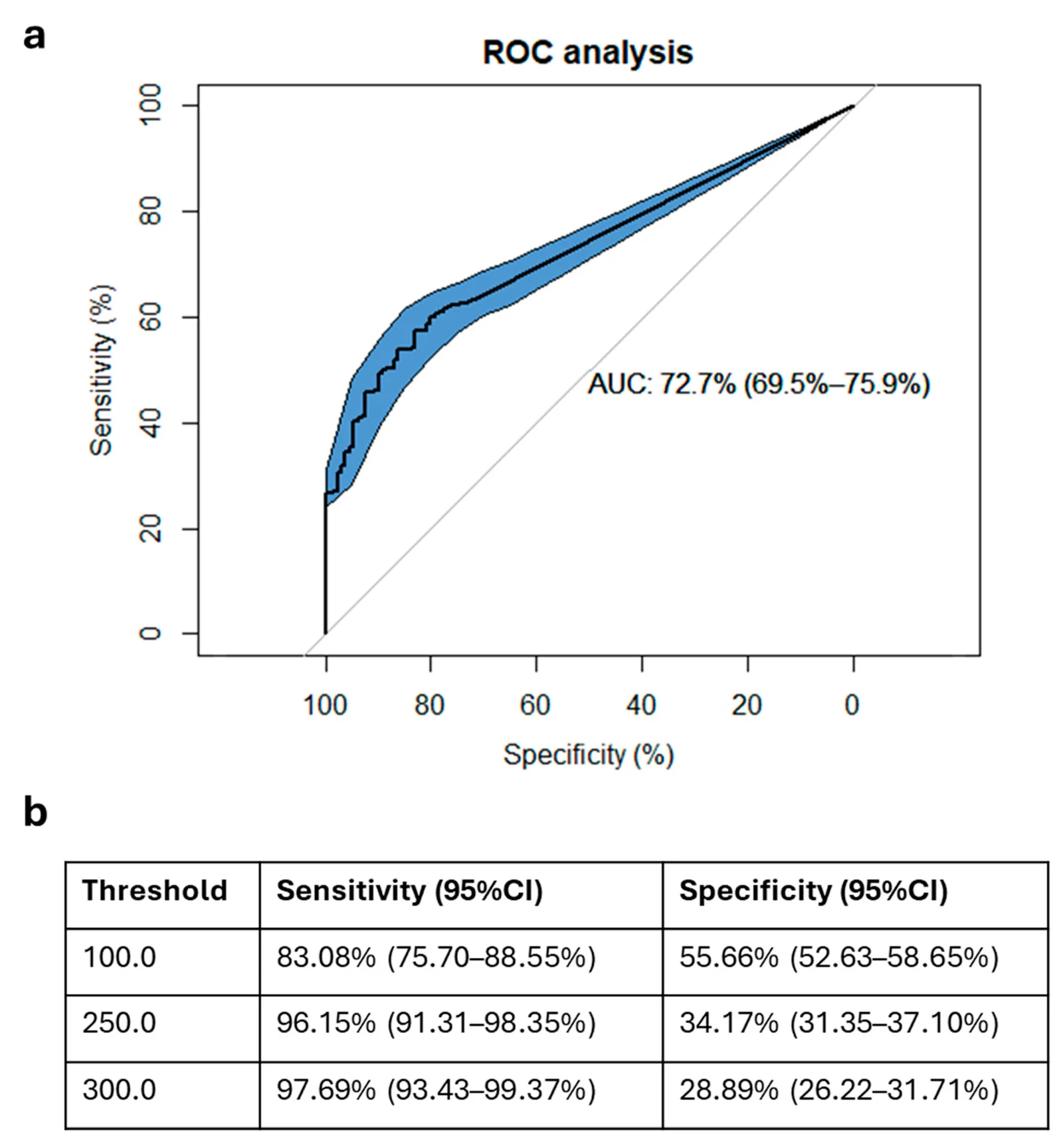
(**a**)—ROC analysis of KREC values in the group of patients with confirmed diagnosis (Case group) and the Control group (95% CI is shown in blue); (**b**)—sensitivity and specificity of different KREC cut-offs in the analyzed cohort.

ROC analysis was performed to discriminate AGG patients from Control group newborns (Figure 1). This analysis yielded a second PCR KREC cut-off value of 248.1 copies per 10^5^ cells, with a likelihood ratio of 8.983.

**Figure 2:**
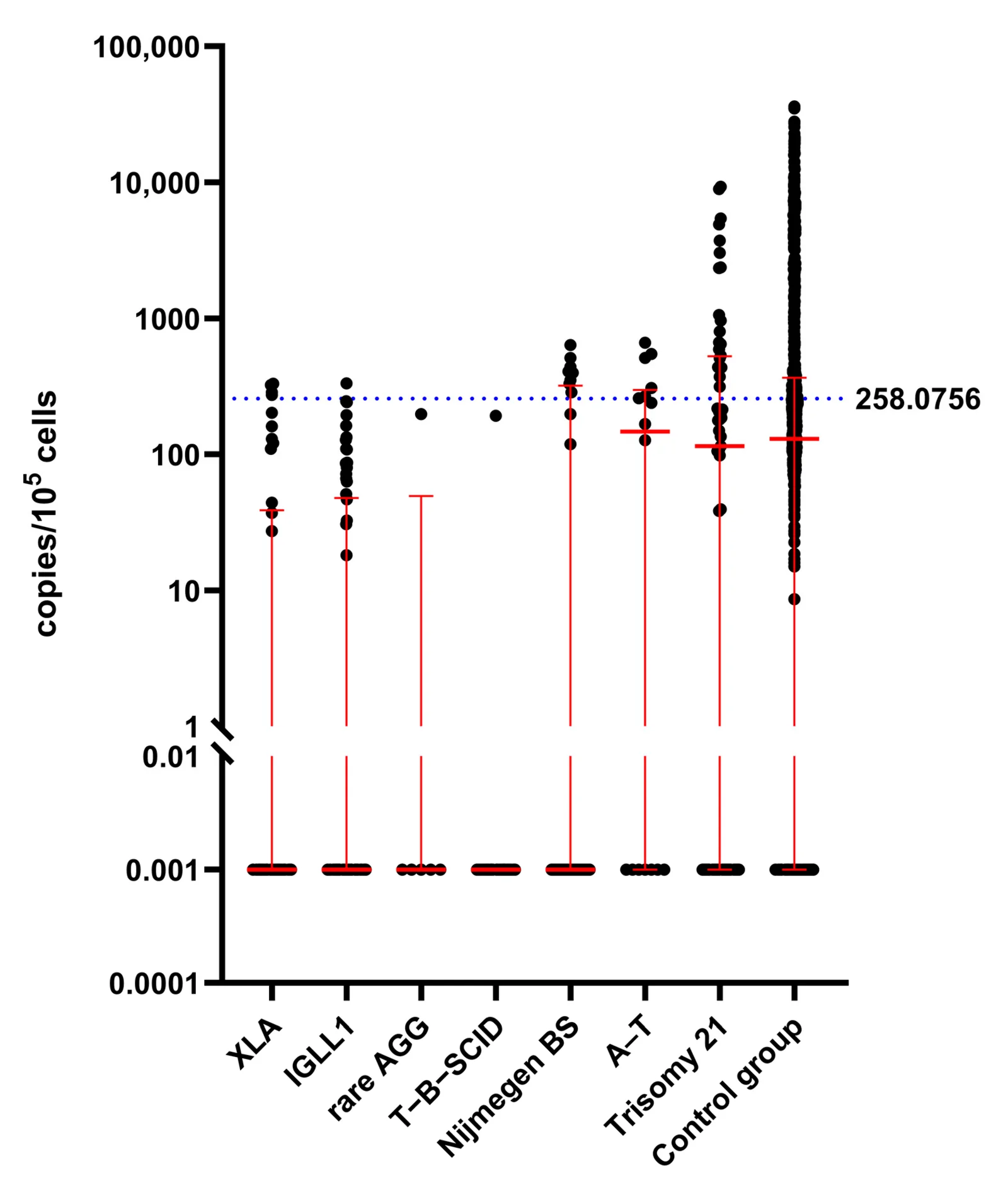
KREC values distribution in different groups of newborns with confirmed diagnosis (Case group) and the Control group. Red lines represent the median and interquartile range. The dotted line represents the cut-off value of concentrations of KREC. Note the log_10_ scale on the Y axis.

Independent analysis of the full cohort of KREC measurements from Cases and Control patients resulted in a cut-off value of 258.0756 copies per 10^5^ cells (95% CI by the delta method: 231.1482–285.0029; 95% CI by 10,000 bootstrap percentile analysis: 198.5407–308.4291) (Figure 2).

During cut-off validation in the Comparison group, our data indicate that 3.03% of measurements in patients with T^−^B^−^ SCID (n = 1/33; 95% CI: 0.08–15.76%) may have KREC values above 100 copies per 10^5^ cells when this cut-off was applied (Table 1, Figure 1). Similar findings were observed in 18.75% of measurements in A–T patients (n = 3/16), 7.14% of Nĳmegen syndrome patients (n = 2/28), and 21.67% of patients with trisomy 21 (n = 13/60). All of these groups would benefit from an increased KREC cut-off. Notably, 4 of 14 Nĳmegen syndrome patients (28.57%; 95%CI: 8.39–58.10%) and 4 of 8 patients with A–T (50.00%; 95%CI: 15.70–84.30) were detected exclusively due to decreased KREC levels, while their TREC values remained above the established cut-off.

In the retrospective pilot NBS cohort (n = 202,908) validation, using a second PCR test KREC cut-off value of 100 copies per 10^5^ cells, the proportion of screening-positive samples at the first PCR stage was estimated at 1.30‰ (95% CI: 1.14–1.47‰). Increasing the cut-off to 250 copies per 10^5^ cells resulted in a 2.1-fold increase in the screening-positive group, to 2.75‰ (95% CI: 2.52–3.00‰). In addition, an extra 0.62‰ of newborns (95% CI: 0.51–0.74‰) were expected to fall into the borderline interval with KREC values of 250–300 copies per 10^5^ cells.

**Figure 3:**
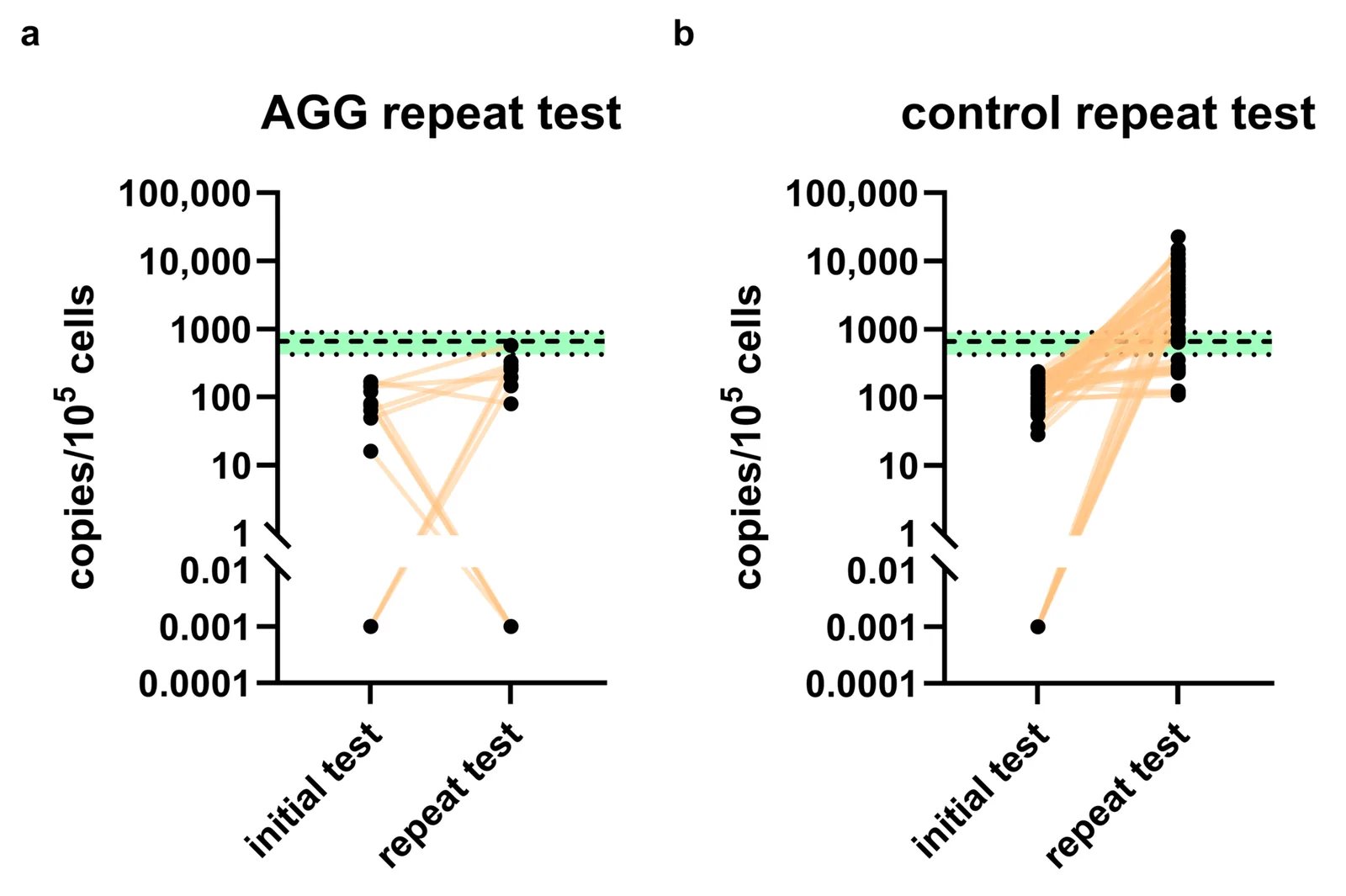
KREC values in patients with AGG diagnosis (**a**) compared with the Control group (**b**). The dotted line represents the cut-off value of concentrations of KREC with 95%CI (shown in green color) at the proposed third PCR stage. Note the log_10_ scale on the Y axis.

Such an increase in the burden of samples requiring confirmatory diagnostics makes it necessary to introduce additional steps to distinguish true AGG cases from false-positive results. We hypothesized that a third PCR performed 1–2 months later could be effective for this purpose. To test this hypothesis, we analyzed repeat KREC measurements in patients with confirmed AGG (typically performed within the first 1–2 months of life) as well as in newborns who were followed up with transient B-cell lymphopenia (TBL). In all cases of TBL, no clinically significant variants on whole-exome sequencing were found, and B cells increased to normal ranges. In total, we included 12 patients with confirmed AGG and 52 newborns with TBL and compared the dynamics of KREC values on repeat testing performed 1–2 months after initial immunophenotyping. Initial KREC values did not differ significantly between the two groups (*p*-value = 0.1750, Mann–Whitney test), whereas repeat KREC measurements in AGG patients were substantially lower than those observed in the Control group (*p*-value < 0.0001, Mann–Whitney test) (Figure 3).

We subsequently performed ROC analysis to identify a threshold capable of discriminating KREC values on repeat testing between AGG patients and newborns with transient B-cell lymphopenia who showed recovery. This analysis was conducted using the thres2 function from the *ThresholdROC* package in R. Analysis of the full cohort of repeat KREC measurements yielded an optimal cut-off value for the third PCR of 655.8737 copies per 10^5^ cells (95% CI by the delta method: 458.0578–853.6896; 95% CI by 10,000 bootstrap percentile analysis: 414.2312–846.5529) (Figure 3). This value is consistent with the first percentile of the KREC distribution in the general population (662.7 copies per 10^5^ cells), as determined in our pilot NBS study [6].

When this threshold was applied at the third PCR stage, only 7.14% of samples (95% CI: 1.50–19.48%) that had fallen below the cut-off at the second PCR stage would be referred for immunophenotyping and subsequent confirmatory diagnostics, and would ultimately be considered normal. Accordingly, a repeat KREC value below approximately 850 copies per 10^5^ cells appears to represent a reasonable cut-off for referral to confirmatory testing when repeat analysis is performed 1–2 months after the second PCR stage. This approach would substantially reduce the burden on both flow cytometry facilities and tertiary immunology units.

## 4. Discussion

This study demonstrates the benefits of adjusting second PCR test KREC cut-off values to improve the sensitivity of newborn screening (NBS) for agammaglobulinemia (AGG) and other primary immunodeficiencies (PIDs) associated with B-cell lymphopenia. By analyzing a large cohort of newborns screened in Russia and applying ROC analysis, we showed that using a KREC cut-off of 100 for first PCR testing and then a KREC cut-off of 250 copies per 10^5^ cells for a second PCR test taken 15–25 days later substantially improves the detection of both classical and atypical X-linked agammaglobulinemia (XLA), other forms of AGG, and additional syndromic PIDs characterized by B-cell lymphopenia. Our findings also highlight the presence of mild AGG phenotypes, which may present with KREC values close to or exceeding conventional thresholds yet still require clinical evaluation and intervention. Collectively, these observations underscore the need for flexible NBS criteria that can accommodate the clinical and genetic heterogeneity of AGG and enable early identification and management of syndromic patients who frequently require immunological monitoring or treatment.

At the same time, we acknowledge the high false-positive rates associated with KREC-based detection in NBS. To reduce the number of unnecessary referrals and mitigate potential negative perceptions of NBS among parents, we propose a revised screening algorithm that incorporates additional dried blood spot sampling and a third PCR step with its own KREC threshold. This approach allows for discrimination between transient B-cell lymphopenia and true-positive AGG or other PIDs associated with B-cell lymphopenia, without adversely affecting clinical outcomes. Importantly, the prolonged asymptomatic window observed even in severe forms of B-cell lymphopenia, such as XLA, provides sufficient time for an extended diagnostic pathway while preserving the benefits of early detection.

Traditionally, it has been assumed that, because NBS uses KREC primarily to target XLA—in which KREC levels are expected to be undetectable or extremely low—a low first and second PCR cut-off value is preferable in order to minimize the high false-positive rate associated with higher thresholds. However, our analysis demonstrates that even patients with XLA may exhibit KREC values exceeding 100 copies per 10^5^ cells when a second PCR test is done after first testing. For example, in our NBS study, 6 of 24 patients demonstrated KREC values exceeding 100 copies per 10^5^ cells at the second PCR test stage. Notably, their first PCR results were <50 copies per 10^5^ cells.

Thus, a substantial proportion of XLA patients exhibited relatively high KREC levels at the second PCR stage, reflecting considerable variability in KREC values within this key subgroup of patients with primary B-cell lymphopenia. Without increasing the KREC screening threshold, at least at the second PCR test stage, these patients would likely have been missed. Such cases would experience delayed diagnosis, potentially approaching the pre-NBS era, when the median age at XLA diagnosis was as high as 28 months, with a reported range of 0–141 months [26].

Specifically, increasing the second PCR test KREC threshold to 250 copies per 10^5^ cells is expected to result in a substantial improvement in sensitivity for the detection of AGG patients. For example, an initial cut-off value of 100 copies per 10^5^ cells allowed for identification of 78.26% of XLA patients (95% CI: 63.64–89.05%), whereas increasing the threshold to 250 copies per 10^5^ cells would enable detection of an additional 13.04% of patients (95% CI: 4.94–26.26%). Notably, 8.70% of XLA patients (95% CI: 2.42–20.79%) demonstrated KREC values exceeding 250 copies per 10^5^ cells. In rare *TCF3*- or *IGHM*-associated forms of AGG, 16.67% of patients (95% CI: 0.42–64.12%) exhibited KREC values above the threshold of 100 copies per 10^5^ cells. As briefly reported previously [19], KREC measurement is critical for the detection of certain cases of “leaky” T^−^B^−^ SCID. One illustrative example is a female patient born at 39 weeks of gestation with a birth weight of 3298 g (Patient 20080624 from [19]), who demonstrated KREC values of 0 copies per 10^5^ cells, while TREC values were 215 copies per 10^5^ cells at the second PCR test stage of the nationwide NBS program. Owing to the absence of KREC, the patient was referred for immunophenotyping, which revealed a marked reduction in CD3^+^CD4^+^ lymphocytes (147 cells/µL, naïve subset, 35.2%), CD3^+^CD8^+^ lymphocytes (83 cells/µL; naïve subset, 45.5%), and CD19^+^ lymphocytes (6 cells/µL; 2.1% of lymphocytes). Based on these findings, the infant was referred for whole-exome sequencing, which identified two compound heterozygous variants in the *ADA* gene, NM_000022.4:c.[792G>A];[845G>A], p.[(Trp264*)];[(Arg282Gln)], consistent with ADA-SCID. Additional cytogenetic analysis revealed a 47,XXX karyotype (trisomy X, also known as triple X syndrome). Trisomy X is associated with highly variable clinical manifestations, ranging from no apparent symptoms to significant disability [33]. The diagnosis of ADA-SCID was subsequently confirmed by reduced ADA enzymatic activity (2.54 nmol/h/mg; normal range: 13.22–36.39) measured in DBS.

These clinical cases illustrate that KREC values in cases of B-cell lymphopenia may exhibit substantial variability, as previously documented in assay validation studies [34]. This variability highlights the importance of defining cut-off values that are sufficiently inclusive to accommodate such fluctuations and ensure reliable identification of at-risk newborns. Appropriately inclusive cut-offs help account for analytical variability and assay limitations, thereby reducing the risk of missed diagnoses, particularly in patients with the above cut-off or borderline KREC levels.

Based on our analysis, a KREC cut-off value of 250 copies per 10^5^ cells appears to be the optimal operational value for newborn screening at the second PCR test stage. Moreover, KREC values within the range of 250–310 copies per 10^5^ cells may be regarded as a borderline interval that warrants interpretive caution and, when clinically justified, repeat assessment; however, this range should not be considered equivalent to the formal screen-positive cut-off requiring immediate referral to the full confirmatory diagnostic pathway.

Analysis of the retrospective cohort from a previously published KREC pilot [6] showed that 2.75‰ of newborns (95% CI: 2.52–3.00‰) could be classified as screening-positive based on second PCR KREC measurement with a cut-off of 250 copies/10^5^ cells. When extrapolated to the national scale, this corresponds to as many as approximately 3300 dried blood spot (DBS) samples per year (95% CI: 3000–3600) requiring referral for confirmatory diagnostics according to the established algorithm. This figure represents nearly a twofold increase compared with the current KREC-based referral rate within the Russian NBS program [19]. Such an increase would be expected to impose a substantial additional burden on flow cytometry facilities and tertiary immunology centers.

Other studies have also reported high false-positive rates associated with KREC-based screening and have proposed additional testing steps to mitigate this burden [35] or, in some cases, have even reconsidered the routine use of KREC in NBS programs [36].

However, it should be taken into account that newborns with AGG, including those with XLA as the most severe forms of B-cell deficiencies, are typically born clinically healthy, without overt signs of impending disease, and do not develop recurrent infections until at least 6–8 months of age, when maternally derived antibodies are no longer protective [37]. Accordingly, the healthcare system has sufficient time to implement repeat testing in newborns with initially low KREC values without compromising clinical outcomes. This approach could represent a practical solution to the high false-positive rate associated with KREC measurement and the consequent increase in referral rates. This hypothesis is supported by the observation by King et al. that KREC levels remain low or undetectable throughout life in patients with XLA [38].

**Figure 4:**
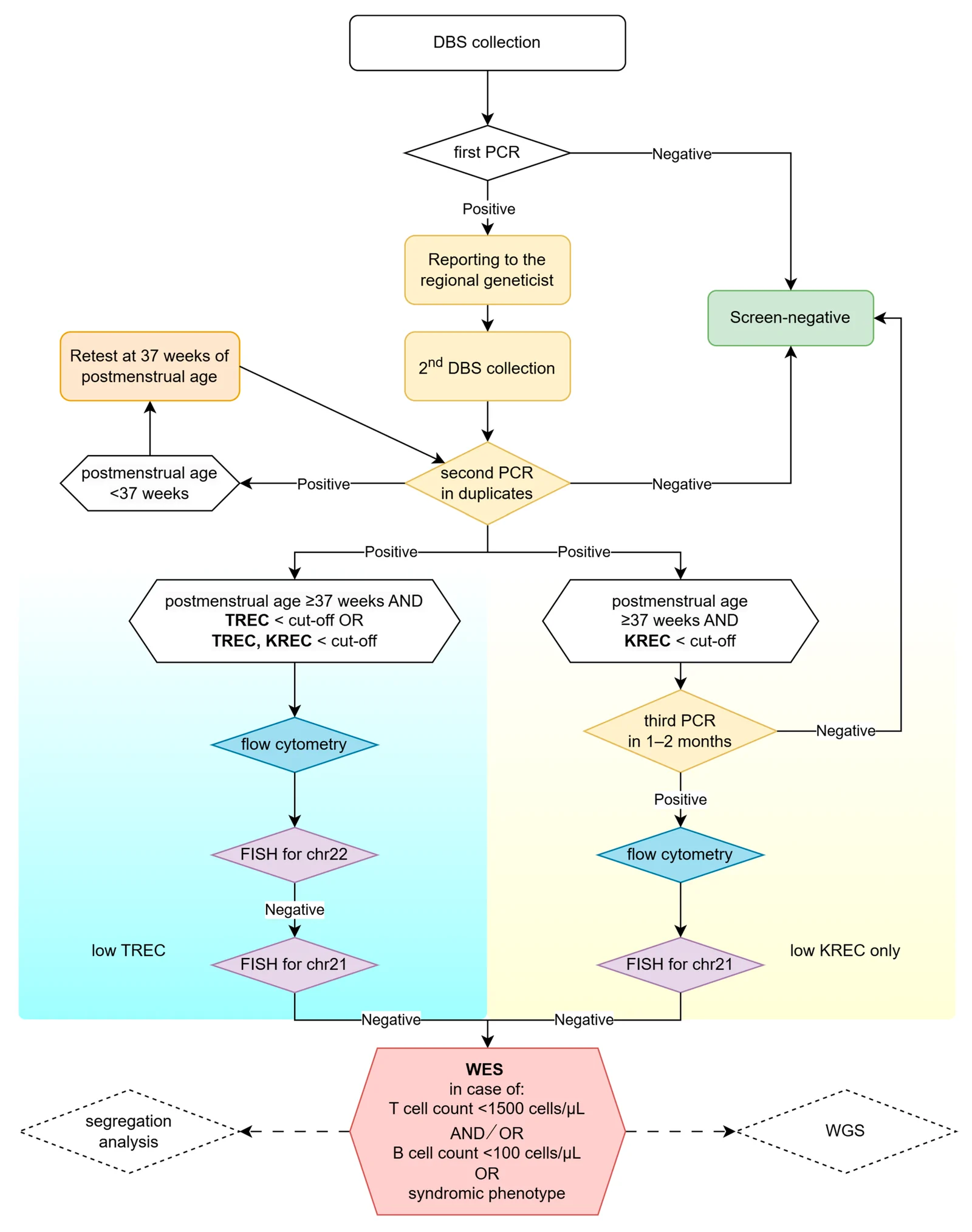
The renewed NBS algorithm implementing third PCR for the KREC screening-positive group of newborns. DBS—dried blood spot; WES—whole-exome sequencing; WGS—whole-genome sequencing.

Thus, we propose an improved three-step algorithm for the management of newborns classified as screening-positive based on KREC measurement (Figure 4). This revised algorithm incorporates an additional step for newborns with KREC values below the proposed new cut-off of 250 copies per 10^5^ cells after the second PCR test. Specifically, it includes repeat KREC measurement from DBS during a third PCR test. Only newborns with repeat KREC values below 850 copies per 10^5^ cells at the third PCR would then be referred for further evaluation, consisting of immunophenotyping by flow cytometry and subsequent genetic testing. We propose scheduling the third PCR test to coincide with the routine pediatric examination at 1–3 months of age, concurrently with vaccination using inactivated vaccines.

The advantages of the revised algorithm are as follows: (i) reduction in the number of false-positive cases that would otherwise proceed through the full diagnostic workflow but ultimately represent transient lymphopenia; (ii) optimization of the NBS workflow by reducing the burden on flow cytometry facilities and tertiary immunology centers; and (iii) use of dried blood spot sampling on Guthrie cards instead of larger volumes of whole blood collected in EDTA tubes requiring urgent transport to flow cytometry laboratories, which is less invasive for infants and psychologically more acceptable for their parents.

Only a limited number of potential disadvantages of the proposed algorithm can be anticipated. These include suboptimal compliance of healthcare providers and families with the collection of an additional dried blood spot sample one or two months later, when the family has already been discharged from the hospital. In real-world practice, we encountered this challenge even at the second PCR test stage, where only 86.54% of samples were successfully collected (95% CI: 85.79–87.27%), while the remaining cases were lost to subsequent diagnostic follow-up [19]. A systematic analysis of the reasons for missing samples is beyond the scope of the present study; however, based on our observations, the most likely contributing factor includes non-compliance with the established NBS workflow by regional screening specialists. We believe that the assessment of missed cases relates primarily to the domain of national public health management rather than clinical diagnostics and thus falls outside the objectives of this manuscript.

Patients with syndromic forms of B-cell lymphopenia clearly benefit from detection through NBS. As demonstrated in the present study, a substantial proportion of patients with ataxia–telangiectasia and Nĳmegen syndrome can be identified exclusively through KREC-based screening. Moreover, low KREC levels have previously been shown to correlate with disease severity in Nĳmegen syndrome [39]. Accordingly, identification of markedly reduced KREC values in these infants may inform early clinical decision-making, including consideration of timely HSCT within the first year of life [39,40,41].

## 5. Conclusions

In conclusion, evidence-based adjustment of KREC cut-off values, combined with third repeat DBS testing, may improve the sensitivity of newborn screening for AGG and other PIDs associated with B-cell lymphopenia while maintaining a manageable referral burden. The proposed algorithm warrants further prospective validation within the nationwide NBS program.

## Figures and Tables

**Table 1:** Second PCR test KREC values in different patient groups.

Patients’ Group	Case Group			Comparison Group				Control Group
Diagnosis	XLA	λ5 Deficiency	Rare AGG	T^−^B^−^ SCID	Nĳmegen Syndrome	A–T	Trisomy 21	
Number of patients	24	39	3	22	14	8	31	534
Number of KREC measurements	46	78	6	33	28	16	60	1042
KREC value (copies/10^5^ cells)								
Minimum	0	0	0	0	0	0	0	0
25% Percentile	0	0	0	0	0	0	0	0
Median	0	0	0	0	0	147.7	115.3	130.3
75% Percentile	39.00	47.83	49.50	0	320.5	298.9	528.0	367.4
Maximum	331.0	333.4	198.0	192.8	638.7	665.0	9315	36,187
Range	330.9	333.4	198.0	192.8	638.7	665.0	9315	36,187
5% Percentile	0	0	0	0	0	0	0	0
95% Percentile	311.9	196.8	198.0	57.85	582.6	665.0	5423	8182
95% CI of median								
Actual confidence level	97.41%	96.92%	96.88%	96.49%	96.43%	97.87%	97.27%	95.00%
Lower confidence limit	0	0	0	0	0	0	0	113.6
Upper confidence limit	0	0	198.0	0	197.7	309.2	214.0	151.2
Coefficient of variation	200.2%	198.0%	244.9%	574.4%	151.6%	115.0%	232.4%	271.3%
Adjusted *p* Value (Dunn’s multiple comparisons test): XLA vs. groups	n/a	>0.9999	>0.9999	0.9743	>0.9999	0.1938	0.0015	<0.0001

n/a—not applicable, as this represents a non-informative comparison within the same group.

**Table 2:** Immunological and genetic characteristics of XLA patients with second PCR test KREC values above 100 copies per 10^5^ cells.

Patient’s Number	TREC at First PCR Stage, Copies/10^5^ Cells	KREC at First PCR Stage, Copies/10^5^ Cells	Average TREC at Second PCR Test Stage, Copies/10^5^ Cells	Average KREC at Second PCR Test Stage, Copies/10^5^ Cells	CD3^+^ Cell Count, Cells/µL	CD19^+^ Cell Count, Cells/µL (% of Lymphocytes)	*BTK* Variant (NM_000061.3) According to the cDNA	*BTK* Variant (NM_000061.3) According to the Protein
1	1166	23	9156	120	3610	0 (0.1%)	ex6–7dup	p.(?)
2	1002	0	2093	163	4306	11 (0.2%)	c.1751-2A>G	p.(?)
3	1482	43	4424	267	4747	46 (0.9%)	c.240G>A	p.(Pro80=)
4	775	21	1381	144	2524	50 (1.7%)	c.319G>T	p.(Asp107Tyr)
5	1163	0	2441	192	3541	22 (0.5%)	c.391+1del	p.(?)
6	610	9	2704	141	3793	20 (0.5%)	c.1922G>A	p.(Arg641His)

## Data Availability

The data that support the findings of this study are available on request from the corresponding author. The data are not publicly available due to privacy or ethical restrictions.

## Abbreviations

The following abbreviations are used in this manuscript:

A–T	Ataxia–telangiectasia
ADA	Adenosine deaminase
AGG	Agammaglobulinemia
DBS	Dried blood spot
FISH	Fluorescent in situ hybridization
IEI	Inborn errors of immunity
KREC	Kappa-deleting recombination excision circles
NBS	Newborn screening
PCR	Polymerase chain reaction
PID	Primary immunodeficiency
SCID	Severe combined immunodeficiency
TBL	Transient B-cell lymphopenia
TREC	T-cell receptor excision circles
WES	Whole-exome sequencing
WGS	Whole-genome sequencing
XLA	X-linked agammaglobulinemia
